# The combined effects of sidestream smoke extracts and glycated serum albumin on endothelial cells and platelets

**DOI:** 10.1186/1475-2840-9-28

**Published:** 2010-07-06

**Authors:** David A Rubenstein, Blake E Morton, Wei Yin

**Affiliations:** 1 School of Mechanical and Aerospace Engineering, Oklahoma State University, Stillwater, USA

## Abstract

**Background:**

The purpose of this study was to test the hypothesis that sidestream tobacco smoke extracts would inhibit the culture of endothelial cells and enhance platelet aggregation under diabetic vascular conditions. Sidestream tobacco smoke and advanced glycation end products are known cardiovascular risk factors and we aimed to determine the combined interaction between these two risk factors to promote cardiovascular diseases associated with diabetes.

**Methods:**

Human umbilical vein endothelial cells were cultured in the presence of sidestream tobacco smoke extracts (SHS) or nicotine and glycated albumin (AGE) or non-glycated albumin. After 3 days, endothelial cell viability and density were investigated. Platelets were also incubated with these compounds for up to 6 hours. Platelet aggregation and the surface expression of CD41 and CD62P were examined. In some experiments, platelets were added to the endothelial cell culture to determine if an interaction between platelets and endothelial cells occurs that can alter the responses to SHS or AGE.

**Results:**

In general, the endothelial cell culture conditions were reduced in the presence of AGE and SHS. Nicotine, did not play a role in this reduction. Platelet aggregation proceeded faster in the presence of AGE and SHS. Interestingly, with the combined culture of endothelial cells and platelets, the endothelial cell culture conditions were improved and the platelet functional changes were diminished in the presence of SHS and AGE, as compared with the individual incubations.

**Conclusions:**

Our data suggests that diabetics that are exposed to SHS may have a higher likelihood for cardiovascular disease development through a diminished endothelial cell viability and an increased platelet activity, which are partially mediated by CD41 and not CD62P. This study provides support for an increased cardiovascular risk for diabetic patients that are exposed to SHS. This study also provides a new experimental technique to monitor platelet-endothelial cell interactions.

## Background

Cardiovascular diseases account for the most deaths per year in the Western countries. The risk for cardiovascular disease formation is highly correlated to the exposure to environmental tobacco smoke, as either mainstream (i.e. the smoker) or sidestream (i.e. secondhand from the smoldering cigarette), and to the occurrence of diabetes. In fact, the diabetic vascular system resembles that of many cardiovascular diseases, which we believe facilitates the formation of other cardiovascular diseases[1]. This is predominantly due to the presence of advanced glycation end products within the vascular system. Plasma proteins are especially susceptible to glycation because of their relatively low turn-over rate and the likelihood for sugars to accumulate within the blood. Depending on the duration of exposure to the sugars, the glycation extent varies, which partially determines the effect of glycation on protein functions[2,3]. Here, we focus on developing a link between the exposure of endothelial cells and platelets to sidestream tobacco smoke or nicotine in the presence of glycated albumin.

Endothelial cells are the salient cell for many cardiovascular diseases because they interact with compounds that enter the bloodstream and partially regulate vascular permeability, angiogenesis and the initiation of many cardiovascular diseases. Platelets have a direct role in the progression of cardiovascular diseases as well. While the effects of cigarette smoke are multifactorial, it has been previously shown that secondhand smoke (SHS) can enhance tumor formation[4], vascular permeability[5] and inflammatory responses[6], which can all lead to cardiovascular disease formation. Nicotine, which is the primary psychoactive component of cigarettes, can enhance the surface expression of some endothelial cell inflammatory mediators[7] and can accelerate some cardiovascular disease formation[8]. Platelet activation and aggregation are also enhanced after exposure to secondhand smoke[9,10], whereas nicotine strongly inhibits platelet activation[10,11].

It has become apparent that advanced glycation end products (AGEs) can regulate many cardiovascular disease processes through changes in endothelial cell and platelet function. AGEs can promote monocyte migration across endothelial cells[12]. The permeability of the endothelial cell membrane[13], the activation/functionality of endothelial nitrous oxide synthase[14] and the transformation of the endothelial cell membrane from anti-coagulant to pro-coagulant are all regulated by the presence of AGEs. Platelet activation, aggregation and expression of cell surface molecules are also influenced by the presence of advanced glycation end products[15]. In regards to both endothelial cells and platelets, the glycation extent (i.e. how many glucose molecules are associated with each albumin) has a strong role on the measured functional changes.

In this study, we investigated the changes in platelet activation, aggregation and cell surface marker expression in response to the combined exposure to advanced glycation end products and sidestream tobacco smoke extracts or nicotine. Endothelial cell culture conditions were also investigated after exposure to advanced glycation end products and sidestream tobacco smoke extracts or nicotine. The combined culture of endothelial cells and platelets was used to investigate if any feedback mechanisms between endothelial cells and platelets exist. We hypothesized that the combined effects of advanced glycation end products and sidestream tobacco smoke extracts on endothelial cell or platelet function would 1) instigate cardiovascular disease pathologies and 2) would be more detrimental to endothelial cells and platelets as compared to the individual exposures. Also, the addition of pure nicotine in the presence of advanced glycation end products would inhibit cardiovascular disease pathologies in both endothelial cells and platelets.

## Methods

### Sidestream Tobacco Smoke Extraction

High tar Marlboro 100's (16 mg tar, 1.2 mg nicotine, Phillip Morris) were used to make sidestream extracts as described previously[10] with the following modifications. Puffing was mimicked by alternating the inflow flow rate between 100 mL/min for 25 seconds to 600 mL/min for 5 seconds. This procedure was followed until the cigarette had burned to ~2 mm of the filter, which took ~10 minutes. Sidestream tobacco smoke was collected in HEPES buffered saline (130 mM NaCl, 20 mM HEPES, pH 7.4) via a step-down manifold to increase extraction efficiency (all chemicals from Sigma-Aldrich, St. Louis, MO unless otherwise noted). For this type of extraction, no smoke can pass through the filter and all smoke was collected from the smoldering end of the cigarette[10].

### Albumin Glycation

Bovine serum albumin was glycated as described previously[15]. Briefly, 50 mg/mL albumin was incubated with 250 mM D-(+)-glucose, 5 mM phenylmethyl-sulfonyl-fluoride (PMSF, Pierce, Rockford, IL, USA), 2 mg/mL aprotinin, 0.5 mg/mL leupeptin,100 mg/mL penicillin and 100 U/mL streptomycin in phosphate buffered saline (PBS, pH 7.4) at 37°C for 8 weeks. Control albumin samples were incubated for the same time without the addition of glucose. Timed samples were removed and dialyzed extensively against PBS. After an 8 week exposure to glucose, each albumin molecule had an average of 31 glucose molecules associated with it[15]. Albumin samples were aliquoted and frozen at -4°C until use.

### Endothelial Cell Culture

Human umbilical vein endothelial cells (HUVECs) were purchased from ScienCell Research Laboratories as passage one cells and were used between passages two and five. Cells were maintained in endothelial cell growth media supplemented with 5% fetal bovine serum, 1× ECGS, 10 U/mL penicillin and 10 μg/mL streptomycin (all from ScienCell) on 1% gelatin coated flasks. Non-glycated albumin (BSA) or glycated serum albumin (AGE) was added to the culture media at a final concentration of 2 mg/mL[15]. Sidestream tobacco smoke extracts were also added to the culture conditions at a final concentration equivalent to 1 cigarette in 5 L (i.e. the approximate plasma concentration after exposure to one cigarette). In other experiments, nicotine was added at a final concentration of 50 nM, which is the approximate nicotine concentration in a smokers plasma after smoking one cigarette. HUVECs were maintained in these conditions for 3 days. Fresh culture media was exchanged on day 1. HUVECs were initially seeded at a density ranging from 800-1000 cells/cm^2^, after reaching confluence during an earlier passage.

For some conditions, the combined effects of platelets and endothelial cells were assessed. Platelets were added to the culture media at a concentration of 100,000/μL (total culture media volume is 2 mL). Platelets were maintained in a HEPES-modified Tyrode's platelet buffer consisting of 135 mM NaCl, 5 mM glucose, 2.7 mM KCl, 0.5 mM Na_2_HPO_4_, 1 mM MgCl_2 _1 mM trisodium citrate, 0.1% bovine serum albumin and 10 mM HEPES (pH 7.4)[10]. For cell culture experiments that included platelets, culture media was exchanged daily to make sure that the platelets were fresh within the culture (other platelet information can be found in the *Platelets *section); old media and platelets were exchanged with fresh media and platelets. Experimental conditions were mimicked in the new media; SHS, AGE, BSA and/or nicotine were added at the appropriate concentration.

### Endothelial Cell Cytotoxicity Assay

Cell viability and density were quantified with a cell cytotoxicity assay consisting of 2 μM calcein and 4 μM ethidium as described previously[16,17]. Briefly, warmed reagents were exchanged with the cell culture media on day 3, 15 minutes prior to imaging at 37°C. After this association time, images were only taken for ~15 minutes at ~5-7 locations per well. Images were documented with a Nikon TE-2000U inverted microscope interfacing with a Coolsnap fast cooled (ES2) digital camera at 10× (Nikon, Plan Fluor DL, NA 0.3) or 20× (Nikon, Plan Fluor ELWD, NA 0.45) objectives.

### Platelets

Fresh platelet rich plasma (PRP) pheresis packs were purchased from Oklahoma Blood Institute. Washed platelets were obtained via centrifugation of PRP at 1100 × *g *for 9 minutes followed by a resuspension of the platelet pellet in the HEPES-modified Tyrode's platelet buffer[10,15]. For flow cytometry studies, the washed platelet concentration was adjusted to 1 × 10^5^/μL in platelet buffer prior to tobacco smoke/albumin incubation. For aggregation studies, the PRP concentration was adjusted to 2.5 × 10^5^/μL with autologous platelet poor plasma prior to incubation. For all platelet experiments, SHS was added to platelets at a concentration of 1 cigarette/5 L, nicotine was added at 50 nM and albumin was added at 2 mg/mL. Platelets that were not in use were kept at room temperature under gentle agitation.

### Platelet Aggregation and Flow Cytometry

Timed PRP samples were warmed to 37°C in a microcuvette and stirred at 1000 rpm in an optical aggregometer (Chrono-Log Model 490-2D, Havertown, PA). Platelet aggregation was antagonized with 20 μM thrombin receptor agonist peptide 6 (TRAP_6_) and the rate of aggregation and the percent change of aggregation were recorded[15]. The rate of aggregation quantifies the speed with which platelets can participate in aggregation whereas the percent change of aggregation quantifies how many platelets are participating in aggregation.

Timed washed platelet samples were incubated with PE-anti-CD41 (GPIIb) for 30 minutes at room temperature. In separate preparations, washed platelet samples were also incubated with FITC-anti-CD62P (P-selectin) for 30 minutes at room temperature. Platelets were diluted 1:5 in platelet buffer and were then immediately analyzed on a flow cytometer (Accuri C6, Ann Arbor, MI) to quantify the surface expression of GPIIb or P-selectin. Flow cytometry was conducted on platelet samples after the combined incubation with endothelial cells.

### Statistics/Data Analysis

Endothelial cell viability is defined as the number of live cells (calcein positive) divided by the total number of cells (calcein + ethidium positive cells). Endothelial cell density is defined as the total number of live cells divided by the image area (calibrated for each objective). This is normalized by the original seeding density to provide a relative measure of proliferation. Combined, this data provides information regarding the toxicity of the culture conditions. All data (endothelial cells and platelets) were tested for significance using Student's paired *t*-test or ANOVA, as appropriate. Tukey's post-hoc test was implemented to determine which treatments were different, if any. All statistics were performed in the Primer for Biostatistics (v4.0) with α = 0.05. Data was repeated for a minimum of 4 independent times, which would include different pheresis packs for platelet experiments.

## Results

### Endothelial Cell Response

**Figure 1:**
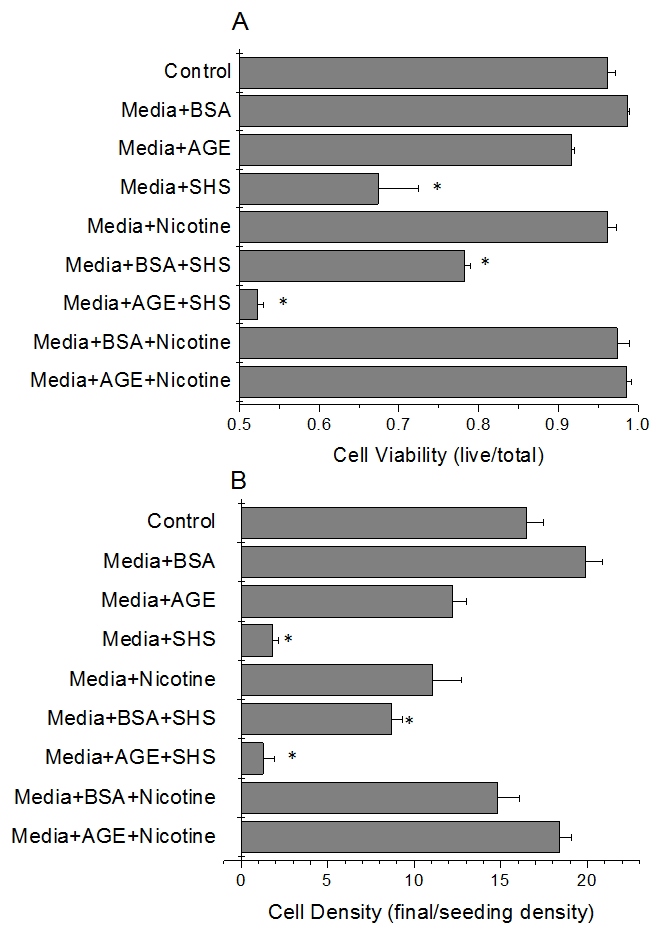
**HUVEC viability (A) and density (B) as a function of cell culture media additive (BSA, AGE, nicotine or SHS)**. Data are the mean + SEM of 7 independent experiments. * differs from control (ANOVA, *P *< 0.05).

To test the endothelial cell response towards the combined effects of secondhand smoke tobacco smoke extracts (SHS) and glycated serum albumin, first we investigated the individual responses to various additives to the cell culture media. Endothelial cell viability and density were measured after 3 days of culture for each additive. With the addition of non-glycated albumin (BSA), glycated albumin (AGE) or 50 nM nicotine to the culture media, the endothelial cell culture conditions were unaffected (Figure 1). However, with the addition of SHS, at a concentration that would be expected to be in blood after exposure to one smoldering cigarette, the culture conditions were significantly lower than the control conditions. In fact, the cell viability approached 60% and the cell density was less than 2 times that of the initial cell seeding density. With any other additive to the culture media, the cell density approached 15 times that of the cell seeding density within 3 days. Therefore, the addition of SHS to endothelial cells can drastically inhibit endothelial cell proliferation.

To address the question posed by this study, we next investigated the effects of SHS exposure under diabetic vascular conditions. For these studies we added SHS (at a concentration equal to the blood plasma level after exposure to one cigarette) in addition to BSA or AGE. The addition of pure nicotine was also investigated to determine the effect of this extract component on the cell culture conditions. With the addition of SHS to either BSA- or AGE-supplemented media the endothelial cell viability and the endothelial cell density were significantly reduced as compared with the control conditions (Figure 1). In fact, the combination of SHS and AGE further reduced the endothelial cell viability and endothelial cell density as compared to either of the additives alone. Again, with the addition of pure nicotine in combination with BSA or AGE to the cell culture media, there was no effect on the cell culture conditions, as compared with control conditions.

### Platelet Response

**Figure 2:**
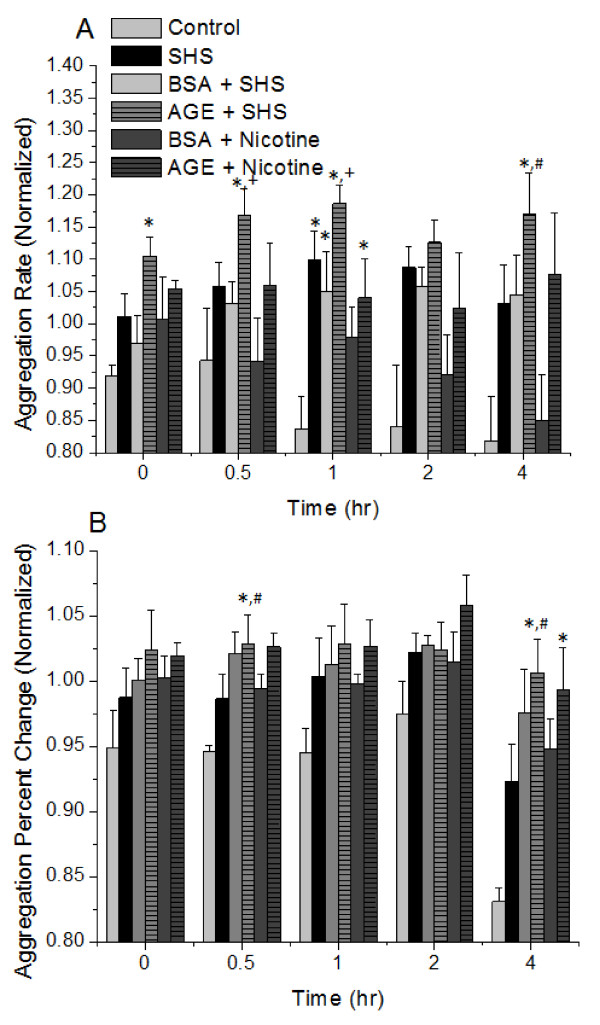
**Platelet aggregation rate (A) and percent change of aggregation (B) in response to second hand tobacco smoke extracts (SHS) and glycated albumin (AGE)**. Data are the mean + SEM of 6 independent experiments. * differs from control (ANOVA, *P *< 0.05, paired by time). + differs from paired BSA (ANOVA, *P *< 0.05, paired by time). # differs from SHS (ANOVA, *P *< 0.05, paired by time).

**Figure 3:**
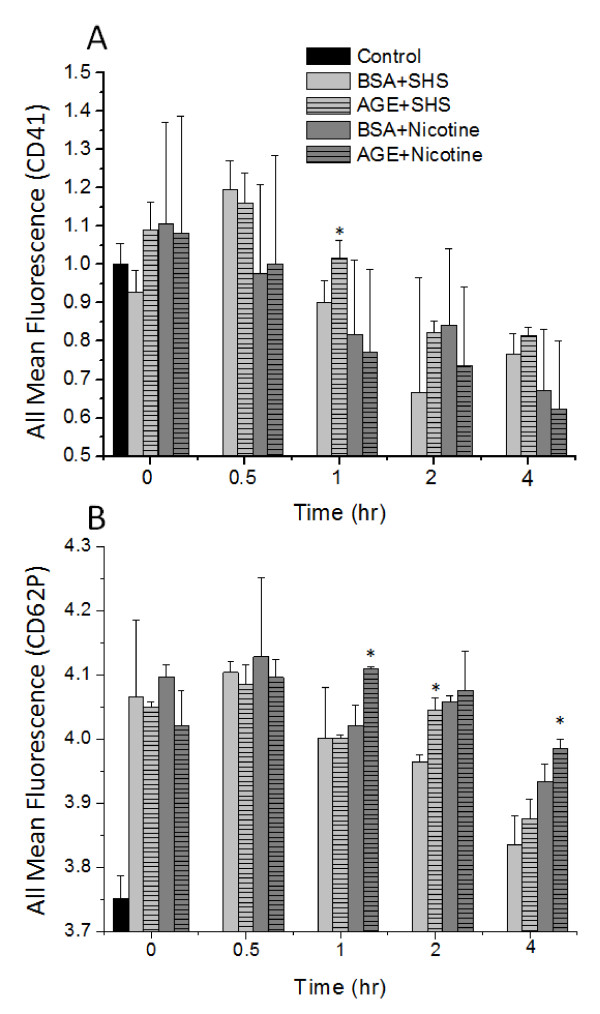
**Surface expression of GPIIb (CD41, A) or P-selectin (CD62P, B) on the platelet membrane after the incubation of SHS or nicotine with AGE or BSA**. Data are the mean + SEM of 4 independent experiments. * differs from incubation with BSA (*t*-test, *P *< 0.05).

The responses of platelets to individual incubations of SHS, nicotine or glycated albumin have previously been reported by us and were therefore not investigated here[10,11,15]. Instead we investigated the combined effects of SHS extract and glycated albumin incubation on platelet aggregation and the surface expression of CD41. Generally, after exposure to SHS extracts the rate of platelet aggregation was enhanced as compared to control samples and the paired non-glycated albumin controls (Figure 2A). With the addition of pure nicotine instead of SHS extracts, there was a general attenuation in the aggregation rate approaching that of control conditions. Interestingly, the percent change of aggregation was generally not affected by the incubation of SHS extracts, nicotine, glycated albumin or non-glycated albumin (Figure 2B). Combined, the aggregation data suggests that platelets that are exposed to SHS extracts aggregate faster, but the quantity of platelets that participate in aggregation remains the same, independent of the culture additive (SHS, AGE or nicotine). Also, nicotine returns that aggregation rate back to control levels, independent of albumin glycation. Flow cytometry was used to confirm the aggregation findings and investigate a possible mechanism for the changes in aggregation rate and percent change of aggregation. In general, the trend in GPIIb expression mimics the data found for the platelet aggregation rate (Figure 3A). With the addition of SHS to the culture media there is an enhanced surface expression of CD41, which was attenuated with the addition of nicotine. We also investigated the expression of CD62P and saw minimal changes to the expression of P-selectin, even in the presence of SHS or nicotine (Figure 3B).

### Combined Endothelial Cell and Platelet Response

**Figure 4:**
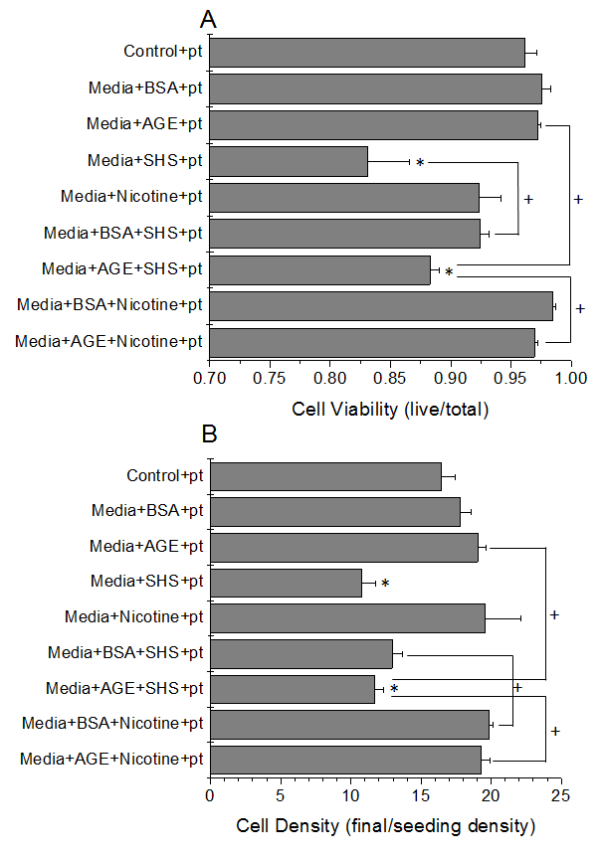
**HUVEC viability (A) and density (B) as a function of combined media additives in the presence of platelets (pt)**. Data are the mean + SEM of 7 independent experiments. * differs from control (ANOVA, *P *< 0.05). + connected groups differ (ANOVA, *P *< 0.05)

**Figure 5:**
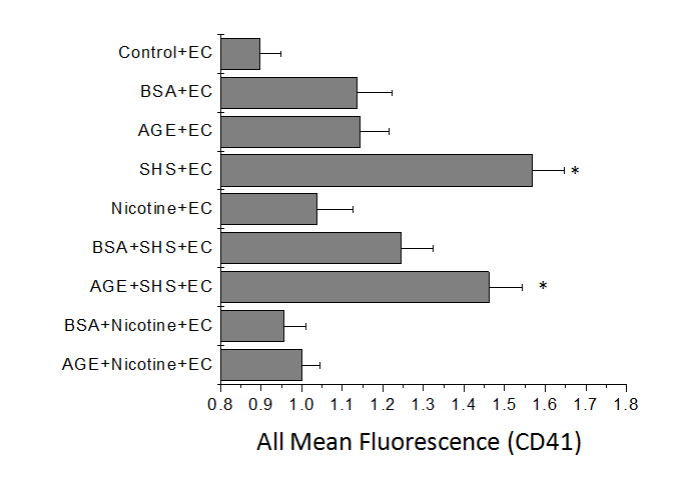
**Surface expression of GPIIb (CD41) on the platelet membrane after the combined incubation of SHS or nicotine with AGE or BSA in the presence of endothelial cells (EC)**. Data are the mean + SEM of 7 independent experiments. * differs from control (ANOVA, *P *< 0.05).

In this study, our aim was to determine the combined effects of SHS and AGE on platelets and endothelial cells. To investigate this, we cultured endothelial cells in the presence of platelets and measured the endothelial cell culture response variables (cell viability and density) as well as the surface expression of GPIIb (CD41) on the platelet surface. In general, with any incubation that included SHS, the endothelial cell viability and cell density was significantly reduced as compared to the control samples (Figure 4). Interestingly, these parameters were slightly enhanced as compared to the endothelial cell incubation without platelets (Figure 1), suggesting that there is some interaction between platelets and endothelial cells which may act to delay cardiovascular disease progression. As before, nicotine did not mediate any of the changes seen here. Platelets followed a similar trend as endothelial cells (Figure 5), where the response measurement for platelets (GPIIb expression), was significantly enhanced after exposure to SHS, but this was generally attenuated as compared with the actions of SHS and AGE on platelets not in the presence of endothelial cells (Figures 3 vs. Figure 5). Combined, the endothelial cell and platelet data suggests that when incubated together, both the endothelial cell responses and the platelet responses are diminished as compared to the responses when the cells are not incubated together.

## Discussion

In this study, we aimed to determine the combined effects of exposure to secondhand tobacco smoke extracts and a diabetic vascular system, induced through the presence of advanced glycated albumin, on endothelial cells and platelets. In previous reports, we have identified that separately, secondhand tobacco smoke extracts and glycated albumin significantly enhance platelet susceptibility to shear induced activation and aggregation[10,15]. Here we started our study by investigating the endothelial cell response to the individual incubation of secondhand smoke extracts or glycated albumin. We then identified the combined responses of these factors on endothelial cells and platelets. Finally, we investigated how these factors affect the combined culture of endothelial cells and platelets. These factors are of interest to us because they are common risk factors for cardiovascular disease initiation/progression and it is critical to identify what effects secondhand tobacco smoke extracts have on a pro-diabetic vascular system (through the presence of glycated albumin) and if endothelial cell culture conditions and/or platelet function change in response to this incubation.

### Endothelial Cell Response

In the first series of experiments, we wanted to verify the effects of sidestream (i.e. secondhand) tobacco smoke extracts or glycated albumin on endothelial cells in culture. With the addition of sidestream tobacco smoke extracts to the culture, we saw a significant reduction in endothelial cell viability and density, which suggests that these tobacco smoke extracts are inhibiting the normal growth/proliferation of endothelial cells in culture (Figure 1). This is in agreement with many other studies on sidestream smoke extracts on endothelial cells that show an increased inflammatory response, a decreased viability and an increased expression of cell membrane receptors, which are common during cardiovascular diseases[18-20]. We also confirmed that the changes in endothelial cell culture conditions were not induced by nicotine, which is the major psychoactive compound in tobacco smoke. In our experiments, the addition of nicotine at a concentration equal to the level found in a smokers blood stream after smoking one cigarette, had no significant effect on endothelial cell culture (Figure 1), which also agrees with the work of others[18]. In fact, it has been suggested that the stimulation of endothelial cell nicotinic receptors enhances cell survival, proliferation and may play an important role in angiogenesis[21]. Others suggest that nicotine can induce cardiovascular diseases by activating endothelial cells[7], which was not investigated here. We also examined the effects of glycated albumin on endothelial cell culture. There were no significant differences between the incubation of endothelial cells with non-glycated albumin and glycated albumin, however, there was a general non-significant reduction in measured variables with the incubation of glycated albumin (Figure 1). Most groups have seen slight changes in endothelial cell function after the incubation with glycated albumin, with no overall hindrance in endothelial cell growth[22,23]. Our findings are in agreement with these studies. Importantly, these initial experiments were conducted to determine the base effects of sidestream tobacco smoke extracts or glycated albumin on endothelial cell culture conditions. Our data suggests that sidestream smoke extracts (SHS) but not pure nicotine, glycated albumin or non-glycated albumin inhibit endothelial cell growth.

To model the effects of sidestream tobacco smoke exposure within a diabetic vasculature, we incubated endothelial cells with sidestream smoke extracts and glycated albumin at the same time. With this combined incubation, the endothelial cell culture conditions were reduced as compared with either incubation by itself, including the incubation of pure sidestream smoke (Figure 1). It was interesting to note that non-glycated albumin seemed to protect the detrimental effects of sidestream smoke to some extent but it could not completely reverse these adverse effects. To the best of our knowledge there have been no previous reports that describe the combined effects of secondhand tobacco smoke extracts and glycated albumin on endothelial cells. We feel that this combined incubation is important to study to begin to elucidate the effects of cigarette smoke exposure (whether mainstream or sidestream) on a diabetic vasculature. Importantly, we saw that combining glycated albumin and sidestream smoke extracts, the effects on endothelial cell culture conditions were dramatically reduced as compared to any of the other conditions investigated here and it seemed to be perpetuated by SHS more so than AGE. We verified that this reduction was not induced by nicotine, which again had no effect on the culture conditions of endothelial cells when incubated with glycated albumin.

### Platelet Response

The platelet response to tobacco smoke extracts, nicotine and glycated serum albumin have been previously reported by us[10,15]. Here, we have extended this work by investigating the combined effects of these compounds on platelet aggregation and the surface expression of CD41. As anticipated, the incubation of platelets with secondhand smoke extracts significantly enhanced the rate at which aggregation precedes (Figure 2). Also, after the addition of nicotine to platelets, the aggregation response returned back to control values. The addition of advanced glycation end products and nicotine had minimal effect on platelet aggregation. All of these findings agrees with prior work from our group and others on the effects of tobacco smoke extracts on aggregation[9,10]. Interestingly, the percent of aggregation was not affected by the combined incubation of SHS extracts, nicotine or glycated albumin. This implies that aggregation proceeds faster but it reaches the same extent of aggregation (i.e. the same quantity of platelets participate in aggregation). Not many groups that we are aware of quantify aggregation in this manner, but in general we saw an enhancement of the aggregation response with SHS incubation, which has been previously reported[9]. This enhancement in aggregation was at least partially mediated by an increase in the expression of CD41 (GPIIb) as measured by flow cytometry (Figure 3A), after exposure to SHS. CD41 is necessary for aggregation to proceed and it would be predicted that with an enhanced CD41 expression aggregation responses would also be enhanced. This also agrees with previous reports that AGE incubation can enhance CD41 expression[15]. In this study flow cytometry measurements were conducted on platelets that were not exposed to shear stress, whereas our previous study[15], flow cytometry was examined after a 40 minute application of shear stress at 10 dynes/cm^2^. We attribute the numerical differences in our percent change data to the shear stress application, however, the trends are still the same; with AGE+SHS incubation, we average a 10-20% increase in CD41 or CD62P expression. It is well documented that shear stress activates platelets and enhances activation markers, such as CD41. Our data would suggest that the combined incubation of SHS and AGE would also enhance CD41 expression, which can potentially mediate increases in aggregation to promote cardiovascular disease formation *in vivo*. In general, we measured no changes in CD62P expression which suggests that the changes we measured with aggregation were not mediated by CD62P. This would also be anticipated because CD62P does not mediate aggregation, however, in other studies[10,11], platelet activation was enhanced after exposure to SHS. Our data suggests that this increase in platelet activation was not mediated by CD62P and that the majority of the changes that we measure are induced by SHS.

### Combined Endothelial Cell and Platelet Response

The major goal of this study was to determine how endothelial cells and platelets interact under diabetic vascular conditions after exposure to sidestream tobacco smoke extracts. To accomplish this, we cultured endothelial cells in the presence of platelets, glycated albumin and cigarette smoke extracts. To the best of our knowledge, no other groups study the combined interactions of endothelial cells and platelets in this pseudo-co-culture system and no groups have investigated how sidestream tobacco smoke can accelerate or decelerate cardiovascular pathologies during diabetic vascular conditions. Under these conditions, we saw that any culture with the addition of sidestream smoke extracts had a reduced endothelial cell viability and cell density (Figure 4). Interestingly, these reductions were somewhat attenuated as compared to the experimental conditions without the addition of platelets. This suggests to us that there is an interaction between platelets and endothelial cells which can inhibit the detrimental effects of sidestream tobacco smoke extracts and/or glycated albumin. Again, we verified that the decrease in cell viability/density was not mediated by nicotine. There are a few additional comparisons that are interesting to point out under these conditions. With the addition of SHS to platelets and AGE there was a significant reduction in endothelial cell viability and density. This suggests that the diabetic conditions we used may not be as detrimental to endothelial cells as expected, but through the addition of SHS, endothelial cells could not tolerate the diabetic vascular conditions. Through the addition of BSA to platelets and SHS, there was a significant improvement in endothelial cell viability and density. *In vivo*, circulating non-glycated albumin may act to protect endothelial cells from altered responses. Also, by substituting between SHS and nicotine, in the presence of platelets and AGE, there was a significant improvement in endothelial cell viability and density. This suggests that nicotine is not the component of SHS extracts that is affecting the endothelial cell culture conditions. It is important to note, that our data suggests that the majority of the effects that we are seeing are mediated by SHS and not AGEs.

Under these same culture conditions, platelets were removed and tested for the surface expression of CD41 (GPIIb), which is necessary but not sufficient for aggregation to proceed. Again, we saw a higher expression of CD41 in the presence of SHS extracts that were incubated with any other compounds (Figure 5). Similar to what we saw with endothelial cells, there was a slight reduction in CD41 expression when incubated with either BSA or nicotine. Compared to the experiments conducted on only platelets, there was a reduction in CD41 expression under these pseudo-co-culture conditions. This again suggests to us, that there is some feedback mechanism between endothelial cells and platelets which helps to minimize pro-cardiovascular disease pathologies. Again, SHS seems to dominate the changes that we are measuring. We were unable to examine platelet functional changes (i.e. platelet aggregation rates) because endothelial cells could not be cultured in the presence of platelet poor plasma and this is required for optical platelet aggregation studies. We are however, optimizing this experimental technique to quantify other platelet functional changes.

## Conclusions

It has previously been reported that tobacco smoke and glycated plasma proteins are associated with an enhanced cardiovascular risk. Here we wanted to investigate the combined effects of SHS extracts and glycated albumin to determine whether or not a diabetic who is exposed to tobacco smoke would have an increased likelihood for the development of cardiovascular diseases. We have seen that after exposure to SHS and AGE, endothelial cells and platelets have a reduced viability/density and a higher likelihood to activate, respectively. This reduced viability and increased aggregation potential was generally heightened as compared with the exposure to either compound (SHS or AGE) individually. Also, with addition of nicotine to either endothelial cells or platelets, the harmful responses were attenuated. Interestingly, when platelets and endothelial cells were cultured together, the reduction in endothelial cell viability and enhancement in aggregation potential, as mediated by changes in CD41 expression, were also attenuated as compared with the individual cell exposure. This would suggest that there is a platelet-endothelial cell feedback mechanism that prevents, to some extent, cardiovascular disease pathologies. However, this pathway has not been identified here, although we have shown that CD41 is partially responsible for the changes that we quantified and CD62P is not responsible for the measured changes. In this work, we have revealed that the combined incubation of SHS and AGE is more potent than the sole incubation of either of these cardiovascular risk factors and this combined incubation may facilitate cardiovascular disease development through a decreased endothelial cell viability/density and an enhancement of the hemostatic system.

## Competing interests

The authors declare that they have no competing interests.

## Authors' contributions

DAR was responsible for the design conception of the experiments, analysis and interpretation of the data, revising the manuscript and the collection of endothelial cell data. BEM was responsible for the collection and analysis of platelet data, the preparation of advanced glycation end products and tobacco smoke extracts and drafting of the manuscript. WY was responsible for revising the manuscript and the analysis/interpretation of the data. All authors have read and approved the final manuscript.

## References

[B1] Harja E Bu DX Hudson BI Chang JS Shen X Hallam K Kalea AZ Lu Y Rosario RH Oruganti S Vascular and inflammatory stresses mediate atherosclerosis via RAGE and its ligands in apoE-/- mice J Clin Invest 2008 118 183 194 10.1172/JCI32703 18079965 PMC2129235

[B2] Monnier VM Stevens VJ Cerami A Maillard reactions involving proteins and carbohydrates in vivo: relevance to diabetes mellitus and aging Prog Food Nutr Sci 1981 5 315 327 6798634

[B3] Cho SJ Roman G Yeboah F Konishi Y The road to advanced glycation end products: a mechanistic perspective Curr Med Chem 2007 14 1653 1671 10.2174/092986707780830989 17584071

[B4] Zhu BQ Heeschen C Sievers RE Karliner JS Parmley WW Glantz SA Cooke JP Second hand smoke stimulates tumor angiogenesis and growth Cancer Cell 2003 4 191 196 10.1016/S1535-6108(03)00219-8 14522253

[B5] Low B Liang M Fu J p38 mitogen-activated protein kinase mediates sidestream cigarette smoke-induced endothelial permeability J Pharmacol Sci 2007 104 225 231 10.1254/jphs.FP0070385 17652909

[B6] Zou N Hong J Dai QY Passive cigarette smoking induces inflammatory injury in human arterial walls Chin Med J (Engl) 2009 122 444 448 19302752

[B7] Ueno H Pradhan S Schlessel D Hirasawa H Sumpio BE Nicotine enhances human vascular endothelial cell expression of ICAM-1 and VCAM-1 via protein kinase C, p38 mitogen-activated protein kinase, NF-kappaB, and AP-1 Cardiovasc Toxicol 2006 6 39 50 10.1385/CT:6:1:39 16845181

[B8] Lau PP Li L Merched AJ Zhang AL Ko KW Chan L Nicotine induces proinflammatory responses in macrophages and the aorta leading to acceleration of atherosclerosis in low-density lipoprotein receptor(-/-) mice Arterioscler Thromb Vasc Biol 2006 26 143 149 10.1161/01.ATV.0000193510.19000.10 16254210

[B9] Folts JD Bonebrake FC The effects of cigarette smoke and nicotine on platelet thrombus formation in stenosed dog coronary arteries: inhibition with phentolamine Circulation 1982 65 465 470 7055868 10.1161/01.cir.65.3.465

[B10] Rubenstein D Jesty J Bluestein D Differences between mainstream and sidestream cigarette smoke extracts and nicotine in the activation of platelets under static and flow conditions Circulation 2004 109 78 83 10.1161/01.CIR.0000108395.12766.25 14691035

[B11] Ramachandran J Rubenstein D Bluestein D Jesty J Activation of platelets exposed to shear stress in the presence of smoke extracts of low-nicotine and zero-nicotine cigarettes: the protective effect of nicotine Nicotine Tob Res 2004 6 835 841 10.1080/1462220042000274284 15700919

[B12] Edelstein D Brownlee M Mechanistic studies of advanced glycosylation end product inhibition by aminoguanidine Diabetes 1992 41 26 29 10.2337/diabetes.41.1.26 1727735

[B13] Li J Schmidt AM Characterization and functional analysis of the promoter of RAGE, the receptor for advanced glycation end products J Biol Chem 1997 272 16498 16506 10.1074/jbc.272.26.16498 9195959

[B14] Bierhaus A Chevion S Chevion M Hofmann M Quehenberger P Illmer T Luther T Berentshtein E Tritschler H Muller M Advanced glycation end product-induced activation of NF-kappaB is suppressed by alpha-lipoic acid in cultured endothelial cells Diabetes 1997 46 1481 1490 10.2337/diabetes.46.9.1481 9287050

[B15] Rubenstein DA Yin W Glycated albumin modulates platelet susceptibility to flow induced activation and aggregation Platelets 2009 20 206 215 10.1080/09537100902795492 19437339

[B16] Rubenstein D Han D Goldgraben S El-Gendi H Gouma PI Frame MD Bioassay chamber for angiogenesis with perfused explanted arteries and electrospun scaffolding Microcirculation 2007 14 723 737 10.1080/10739680701410173 17885997

[B17] Rubenstein DA Venkitachalam SM Zamfir D Wang F Lu H Frame MD Yin W In vitro biocompatibility of sheath-core cellulose-acetate-based electrospun scaffolds towards endothelial cells and platelets Journal of Biomaterials Science, Polymer Edition 2010 in press 10.1163/092050609X12559317149363 20537251

[B18] Argacha JF Fontaine D Adamopoulos D Ajose A van de Borne P Fontaine J Berkenboom G Acute effect of sidestream cigarette smoke extract on vascular endothelial function J Cardiovasc Pharmacol 2008 52 262 267 10.1097/FJC.0b013e318185fa26 18806607

[B19] Gairola CG Drawdy ML Block AE Daugherty A Sidestream cigarette smoke accelerates atherogenesis in apolipoprotein E-/- mice Atherosclerosis 2001 156 49 55 10.1016/S0021-9150(00)00621-3 11368996

[B20] Hutchison SJ Sudhir K Chou TM Sievers RE Zhu BQ Sun YP Deedwania PC Glantz SA Parmley WW Chatterjee K Testosterone worsens endothelial dysfunction associated with hypercholesterolemia and environmental tobacco smoke exposure in male rabbit aorta J Am Coll Cardiol 1997 29 800 807 10.1016/S0735-1097(96)00570-0 9091527

[B21] Jacobi J Jang JJ Sundram U Dayoub H Fajardo LF Cooke JP Nicotine accelerates angiogenesis and wound healing in genetically diabetic mice Am J Pathol 2002 161 97 104 12107094 10.1016/S0002-9440(10)64161-2 PMC1850685

[B22] Kunt T Forst T Harzer O Buchert G Pfutzner A Lobig M Zschabitz A Stofft E Engelbach M Beyer J The influence of advanced glycation endproducts (AGE) on the expression of human endothelial adhesion molecules Exp Clin Endocrinol Diabetes 1998 106 183 188 10.1055/s-0029-1211974 9710358

[B23] Cohen MP Wu VY Cohen JA Glycated albumin stimulates fibronectin and collagen IV production by glomerular endothelial cells under normoglycemic conditions Biochem Biophys Res Commun 1997 239 91 94 10.1006/bbrc.1997.7420 9345275

